# A Letter to Sir B. Brodie, Bart. On the Application of the Collegiate System to the Medical Schools of the Metropolis

**Published:** 1841-10-01

**Authors:** 


### A Letter to Sir B. Brodie, Bart. on the Application of the Collegiate System to the Medical Schools of the Metropolis. By the Rev. *J. H. North*, M.A. Chaplain to St. George's Hospital.

This Letter does honor to the head and heart of its writer, and is worthy of the gentleman to whom it is addressed. There breathes not a man more desirous to ameliorate the condition of medical students, nor more anxious to raise the character of the profession than Sir Benjamin Brodie. His liberality is not confined to words or opinions, but expands into acts of the most generous character. The Pamphlet before us is rightly dedicated to him.

Mr. North displays, in a most effective manner, the comfortless condition of a large proportion of our students. What can be more true than this?

"There is no officer connected with the hospital at which the pupil is entered, whose province it is to guide him in the selection of his apartments; there are no limits within which his choice is confined; there is no warrant for the respectability of the persons at whose house he may take up his abode; there is no provision made for the regularity of his meals, nor for any of those arrangements upon which his comfort depends. He has to settle and arrange for himself all those household affairs, which present no slight difficulty to those who are far older and more experienced than himself. He is alone; and solitude in London is of all things the most desolate. In these circumstances he applies for guidance to those who, not unfrequently, are least able to render him effectual aid; he is at least as likely to fall into bad hands as into good; it may be, a notice stuck up in the hall of the hospital catches his eye, and he takes refuge from his perplexity in the first lodging-house that presents itself; and in this situation, unfriendly to his moral character, unfavourable to study, not admitting any superintendence, and destitute of all domestic comfort, he passes the first months, perhaps, which in the whole of his life have not been spent in the society of his family or friends."

Contrast the neophyte in medicine with the Freshman at College. *There* every arrangement has a direct reference to his advantage. Mr. North proposes to assimilate, as far as possible, the material condition of the medical student and the resident at an University—to provide apartments within the walls of the school—to license lodging-houses for those who prefer them—to establish a common hall and common dinner—and to exercise, as far as possible, a gentle surveillance on the morale as well as the physique of students.

The *enforcement* of regularity, and the adequate adjustment of restraint, are hinted at by contrast.

"I will again recur to the situation of under-graduates in support of this part of my argument. To speak briefly, the means insisted upon to secure regularity of conduct are these: daily attendance at chapel, again at lectures, a third time at hall; and lastly, the system of gates, by which a return of every under-graduate who is out after ten o'clock at night is daily made to the tutor or dean. The punishments for offences against these laws consist of impositions and deprivation of liberty for periods varying in length, and eventually loss of terms and consequent postponement of the degree. I am not aware, that the discipline of our universities has ever been considered too severe; nor do the tutors and deans find that they have too much power for the purpose of maintaining due attention to the necessary points of correct behaviour. Yet of all these restraints—these wholesome and necessary restraints—the life of a medical student is wholly devoid. There is, indeed, some attendance required on lectures, some in the wards of the hospital; but, with this exception, the pupil is entirely his own master; that is, in all matters relating to his hours, his expenses, his companions, his religious and moral habits, he is utterly without a check; and in all the heat and inexperience of youth, he finds all London before him for the uncontrolled gratification of his favourite desires, whatever they may chance to be."

That something *should* be done is manifest—that something *will* be done is not unlikely—and that the *sooner* it is done the better for the profession and the public is, we conceive, undeniable. There will be difficulties, no doubt, in the way. What great and comprehensive scheme of good is free from them? Capital is to be obtained, license to be bartered for restraint, repugnance to Collegiate discipline to be conquered without the aid of prescriptive Collegiate dignity and power—a new machinery to be organised and put in motion. All difficulties, we say, but none insuperable—difficulties that will appear great or trivial as we regard them with the eye of a mere speculator, or of one zealous for men's improvement. For our own part, we believe that those who have originated this excellent plan, and those who will carry it out, deserve the best thanks of the profession, and the good name that crowns benevolent purposes and deeds.

